# Development of the Socioeconomic Screening, Active Engagement, Follow-up, Education, Discharge Readiness, and Consistency (SAFEDC) Model for Improving Transitions of Care: Participatory Design

**DOI:** 10.2196/31277

**Published:** 2022-04-12

**Authors:** Ji Youn Shin, Nkiru Okammor, Karly Hendee, Amber Pawlikowski, Grace Jenq, David Bozaan

**Affiliations:** 1 Department of Media and Information Michigan State University East Lansing, MI United States; 2 Integrated Michigan Patient-Centered Alliance in Care Transitions (I-MPACT) Michigan Medicine Ann Arbor, MI United States; 3 St. Joseph Mercy Ann Arbor, MI United States; 4 Department of Internal Medicine Michigan Medicine University of Michigan Ann Arbor, MI United States; 5 Ann Arbor Veterans Affairs Ann Arbor, MI United States

**Keywords:** care transition, discharge, readmission, patient-centered care, design, participatory design

## Abstract

**Background:**

Transition to home after hospitalization involves the potential risk of adverse patient events, such as knowledge deficits related to self-care, medication errors, and readmissions. Despite broad organizational efforts to provide better care transitions for patients, there are challenges in implementing interventions that effectively improve care transition outcomes, as evidenced by readmission rates. Collaborative efforts that require health care professionals, patients, and caregivers to work together are necessary to identify gaps associated with transitions of care and generate effective transitional care interventions.

**Objective:**

This study aims to understand the usefulness of participatory design approaches in identifying the design implications of transition of care interventions in health care settings. Through a series of participatory design workshops, we have brought stakeholders of the health care system together. With a shared understanding of care transition and patient experience, we have provided participants with opportunities to generate possible design implications for care transitions.

**Methods:**

We selected field observations in clinical settings and participatory design workshops to develop transitional care interventions that serve each hospital’s unique situation and context. Patient journey maps were created and functioned as tools for creating a shared understanding of the discharge process across different stakeholders in the health care environment. The intervention sustainability was also assessed. By applying thematic analysis methods, we analyzed the problem statements and proposed interventions collected from participatory design workshops. The findings showed patterns of major discussion during the workshop.

**Results:**

On the basis of the workshop results, we formalized the transition of care model—the socioeconomic, active engagement, follow-up, education, discharge readiness tool, and consistency (Integrated Michigan Patient-centered Alliance in Care Transitions transition of care model)—which other organizations can apply to improve patient experiences in care transition. This model highlights the most significant themes that should necessarily be considered to improve the transition of care.

**Conclusions:**

Our study presents the benefits of the participatory design approach in defining the challenges associated with transitions of care related to patient discharge and generating sustainable interventions to improve care transitions.

## Introduction

### Improving Transitions of Care to Reduce Hospital Readmission

The US health care system has undergone a drastic shift in the past decade as payment to health systems has transitioned to rewarding value over the volume of care. Quality-based repayment programs incentivize hospitals through payment withholding or incentive-based payments based on outcomes instead of volume. Models such as the Hospital Readmission Reduction Program (HRRP), established in 2012 under the Affordable Care Act, financially penalize hospitals if they have higher than expected risk-standardized 30-day readmission rates [1,2].

Many quality reimbursement models focus on conditions that have a large morbidity and mortality burden, contribute to significant direct and indirect health care costs, and have clear and direct measures to target [3]. Many value-based purchasing programs, including HRRP, focus on improving the treatment of specific disease conditions such as acute myocardial infarction, heart failure, pneumonia, chronic obstructive pulmonary disease, coronary artery bypass graft surgery, elective primary total hip arthroplasty, and total knee arthroplasty [4].

In particular, the HRRP has forced hospitals to recognize that the transition to home after hospitalization carries a significant risk of adverse patient events, readmissions, and increased costs [5]. Transition of care interventions refer to improvements developed to reduce readmission rates among populations transitioning from one care setting to another [6]. Despite ongoing organizational efforts to improve care transitions, there continue to be challenges in implementing interventions that consistently affect key care transition quality indicators such as 30-day readmission rates [7,8].

Although transitions involve multiple stakeholders in the care continuum, including physicians, nurses, hospitals, primary care organizations, and patients, the transition of care interventions were traditionally developed from the health care provider perspective [9]. These interventions are often limited to a single phase in the care continuum, involve only a single institution, and are often limited to interventions considered important from the provider’s perspective [10]. Patients often remain passive participants in the health care system when discussing care transitions. As a result of these hierarchical approaches, developing and implementing effective interventions has been a significant challenge in improving care transitions.

As hospitals and physician organizations (POs) have recognized the benefits of working together to reduce readmissions through programs such as the HRRP [11,12], health care organizations have started to highlight the increased benefits of nonhierarchical collaborative efforts that bring health care professionals, patients, and caregivers together. This collaboration is necessary to identify gaps associated with transitions of care and generate effective interventions.

Health care organizations, private payers, the Centers for Medicare and Medicaid Services, and policy makers have been charged with the task of creating unique ways of incentivizing hospitals and POs to improve patient outcomes and reduce readmissions. An example of this collaborative approach is the Integrated Michigan Patient-centered Alliance in Care (I-MPACT) Collaborative Quality Initiative (CQI) [13]. I-MPACT is a patient-centered CQI that engages hospitals, POs, and patients throughout Michigan and supports the development and implementation of innovative approaches for improving care transitions. The collaborative is composed of numerous health system groups, or clusters, in the Michigan area, which comprise a hospital, a partnering outpatient PO, and patients and caregivers from the local community. Within the CQI, clusters are asked to identify the target population and implement interventions to reduce readmissions for the target population. This initiative offers a unique opportunity for hospitals and PO groups to collaborate to improve care and aligns seamlessly with value-based programs such as the HRRP.

Although the benefits of nonhierarchical collaborative efforts are acknowledged, the lack of effective tools to engage stakeholders often discourages collaborative, multidisciplinary solutions that bring together patients, hospital systems, and outpatient systems [14,15]. As a result, patients’ voices are still marginalized and devalued when identifying problems or creating effective approaches to alleviate readmission [16]. There is a call for effective tools that will help hospitals, associated outpatient organizations, patients, and community caregivers in defining gaps associated with transitions of care.

### Need for a Novel Approach to Identify Gaps Associated With Transitions of Care: Value of Participatory Design

Participatory design is often defined as the involvement of end users of services or products in the early phases of the development process [17-19]. The origins of the participatory design approach are from the 1970s Scandinavian cooperative design movement. When computers were first introduced in the work environment, workers were concerned that they would no longer have the agency to control their tasks and that they would be replaced by newly implemented technologies [17]. To respond to these changes, they aimed to take an active role in managing their work environment. This workplace democracy movement influenced the origin of the participatory design approach and yielded detailed design techniques to invite users throughout the design process [19]. Activity-based methods, including collaborative workshops, brainstorming, and drawing activities, are effective in understanding end users’ expertise and experiences [20,21]. As participatory design has emerged beyond the field of design and human-computer interaction, researchers in other fields, including health care, have started to apply different participatory methods and tools during projects [18,22]. As health care interventions are often designed and implemented from providers’ perspectives, participatory design approaches are considered effective cocreation methods when they include the patient’s perspective.

One of the most widely used participatory design methods is the collaborative design workshop. During the workshops, participants with different perspectives often form subgroups and discuss experiences, insights, and ideas regarding health care interventions through collaborative activities [17,23]. In these collaborative workshops, participants are recognized as knowledgeable stakeholders and experts on particular health issues (eg, disease-specific experiences). Recent health care and human-computer interaction literature has highlighted the benefits of a participatory design workshop with people with special needs in health care contexts (eg, older adults [24], people with dementia [25], pediatric patients, and their caregivers) [24-28]. By participating in different activities, participants can open up conversations regarding complex and unfamiliar social issues during participatory design sessions. For example, Harrington et al [26,29] conducted 2 community-based workshops with underserved populations in the United States. They identified how participants managed their health and the function of design workshops as facilitators for health-related discussions in their communities. Different activities were considered, including mapping activities, to draw participants’ insights into their health management. Similarly, Unbehaun et a [30] conducted participatory, co-design sessions with 14 people with dementia and their caregivers to understand how exergames can be integrated into their daily routines. From multiple design sessions, researchers were able to iteratively modify their design ideas toward users’ motivations and interests. As these examples show, participatory design approaches helped health care researchers develop a deeper understanding of the lived experiences of technology users and engage them in the design of interventions.

To our knowledge, this study is the first to apply participatory design approaches in the context of care transition. As care transitions are a series of events throughout the care continuum rather than a single event, professionals who are engaged in a patient’s care transition typically witness only a part of the whole process. As participants perceive care transitions from multiple perspectives, the authors sought to develop a shared understanding of the care transition. A participatory design workshop was proposed as an effective approach to build a consensus on the care transition processes among different stakeholders.

Building on previous studies that showed the benefits of participatory design methods in different health care settings, this study suggests expanding the application of participatory design workshops beyond a single institution and a single trial. The authors set out to examine the usefulness of inviting multiple health care institutions into the participatory design workshop and the sustainability of the interventions, including long-term follow-up with the participants.

### Research Aims

The overall purpose of this study is to identify the design implications of transition of care interventions derived from the participatory design workshop series. On the basis of qualitative analyses of ideas collected during the workshop, we discuss the major themes of transitional care interventions developed by the project clusters. Our study will contribute to improving methodological innovations in developing a shared understanding of the transition of care that encompasses the patient’s perspective.

## Methods

### Study Design

The goal of this study was to apply participatory design approaches in the context of care transition and generate emerging themes for interventions across different hospitals. We highlight the benefits of gathering stakeholders from a local hospital for collaboration, using patient-centered perspectives, and incorporating participant observation in the hospital setting. During the postworkshop period, we also captured the following: (1) the status of the intervention (active or inactive, modifications, and maintenance), (2) the status of the cluster (participated or dropped out during the study), and (3) whether the cluster conducted an observation. In the *Intervention Sustainability Follow-up* section, we summarize the data collected from 2016 to August 2020.

### Ethical Considerations

I-MPACT’s quality improvement efforts were reviewed by the Institutional Review Board at the University of Michigan (IRB# HUM00126940) and determined to be exempt.

### Data Collection

#### Observation and Patient Journey Maps

Within each cluster, on the day of discharge, a charge nurse identified each patient for observation from the target population. Observations were conducted before discharge from early morning to when the patient physically left the hospital, by an I-MPACT representative not affiliated with the hospital, after obtaining consent from the patient [17]. Observations lasted between 2 and 8 hours and were primarily focused on the patient’s perspective during the process. Timestamps were recorded of patient-provider interactions; patients’ and caregivers’ behaviors; and details surrounding any type of patient instruction or education, including medication administration, self-care activities, mobility restrictions, and wait or idle time. On the basis of the observation data, we created a journey map that shows an overview of each patient’s day of hospital discharge [31]. As a result, 26 unique patient journey maps were created from these observations, with at least one or more patient journey maps from each cluster (Multimedia Appendix 1). The 26 patient journey maps were generated from each participating hospital and shared with all stakeholders at the start of participatory design workshops. These patient journey maps represented a discharge timeline from the patient’s perspective and included all events associated with the discharge process that were considered significant (Multimedia Appendix 1).

#### Participatory Design Workshop

When each cluster joined I-MPACT, they were required to attend a participatory design workshop. This all-day commencement event was intended to bring a cluster together to discuss the local transition of care data and share ideas and insights around the patient’s perspective from various points along the care continuum. Clusters were asked to use this workshop to generate ideas about the problems that the local cluster would target during their participation in I-MPACT over the next several years to improve transitions of care and patient experiences. To create a more collaborative environment and draw rich insights from multiple stakeholders, each commencement event was organized to create a collaborative environment, draw rich insight from multiple stakeholders, and highlight the patient perspective.

A total of 5 participatory design workshops were held between 2016 and 2018 (Table 1). With each cluster attending this commencement event, the aim was to foster discussions where participants could share multiple perspectives, capture the full scope of the care transition process, and generate practical design implications for improvement in care transitions. Each workshop had approximately 40 to 65 participants. During the full-day workshops, participants, comprising patients, caregivers, physicians, nurses, administrators, and designers, worked in groups of 7 to 9 people within their cluster to generate problem statements and begin initial discussions regarding interventions. Each cluster cohort participated in 1 workshop.

**Table 1:** Study cohorts and their commencement dates.

Cohort	Clusters involved in the cohort	Start date
Cohort 1	1 CHF^a^ and 1 SNF^b^	February 2016
Cohort 2	4 CHF	September 2016
Cohort 3	1 SNF and 5 CHF	February 2017
Cohort 4	2 COPD^c^ and 1 CHF	September 2017
Cohort 5	1 CHF and 3 SNF	September 2018

^a^CHF: congestive heart failure.

^b^SNF: skilled nursing facility.

^c^COPD: chronic obstructive pulmonary disease.

At each commencement workshop, participants were provided with patient journey maps created from the observation of a patient discharged from the hospital within their cluster (Multimedia Appendix 2). The details shared from each of the 26 observed discharges focused on the transition of care discussion from the patient’s perspective in an effort to enable participants to gain a better perspective on problems that may otherwise have gone unnoticed by others. Participants also shared their individual experiences regarding care transition, which contributed to a shared understanding of the entire discharge process.

After reviewing the patient journey map and initial discussions, participants were asked to collaboratively generate major problem areas that they aimed to improve through transitions of care interventions. Inspired by service blueprint mapping in service design [32], we encouraged participants to create their own hospital’s discharge timeline that detailed the core activities of a typical patient’s care transition. This process enabled the participants to create a broader representation of the discharge process and consider the major barriers and details that affect a patient’s experience during the care transition (Multimedia Appendix 3). During this process, each group was able to identify barriers, draft problem statements, and generate potential design interventions aimed at improving patient experiences throughout the care transition. If brainstorming was not completed during the workshop, each hospital finalized their brainstormed ideas for transitional care interventions during the postworkshop phase. As a result, 47 original interventions were generated with the aim of facilitating and improving care transitions. Each cluster was required to update its progress in a biannual report called the quality initiative (QI) log, and the August 2020 report was used to assess the status of their interventions. In the *Results* section, we have provided an overview of postworkshop progress to gauge the sustainability of these interventions.

### Data Analysis

We followed a constructivist grounded theory approach for data analysis [33]. Without a predetermined conceptual frame, we iteratively read the participants’ problem statements and identified commonly addressed themes in a collaborative manner. This methodology enabled the team to understand participants’ experiences and views on care transition rather than our views or the preconceptions of health care researchers. To identify the patterns of major discussion during the participatory design workshop, we analyzed problem statements and proposed interventions collected from 79% (19/24) of the hospitals after the workshop. A total of 5 hospitals were not included in the study as 3 (60%) hospitals did not conduct an observation to generate a patient journey map, and the other 2 (40%) hospitals opted out of I-MPACT before August 2020. The lead author (JYS) performed open coding using NVivo Pro 12. While data analysis was performed, the team discussed the general direction and primary focus to create a shared understanding. The team iteratively reviewed the data to identify the major themes for open coding. This resulted in the following topic areas: (1) emerging obstacles during the discharge processes, (2) problem statements that represent the most significant and feasible areas for improving care transitions among identified issues, and (3) generated design opportunities that could be turned into practical interventions. With these themes, we aggregated the problem statements until they did not overlap. This open coding yielded 206 codes, including 106 (51.5%) emerging problems of care transition and 100 (48.5%) opportunities to contribute to interventions. Some examples of coded problems included *lack of patient’s involvement in managing their health* and *lack of consistent scheduling of follow-up appointments*. Opportunities for interventions included the following: *ensure that patient and family caregivers understand education* and *encourage patients to take control over their care*. We used affinity clustering to identify the commonalities and hierarchies of the 206 codes. The lead author (JYS) performed the affinity clustering based on the commonalities and relationships between themes, identifying the most salient emerging themes. The results were presented to the rest of the team to resolve any lack of agreement on the themes. Affinity diagramming resulted in themes with 3 different levels, which allowed the team to capture overarching themes that encompassed the lower-level themes. Themes at the third or lowest level included *patients cannot afford prescriptions* and *differences in the role of social workers and case managers at a hospital.* Second-level themes included *lack of early communication among providers* and *documenting patients’ goals.* Top-level themes included *needs for screening tools* and *the importance of consistency.* This iterative analysis allowed the team to identify themes that workshop participants deemed most critical and recognized as areas needing significant transitions of care improvement.

## Results

### Overview

Six major themes emerged from the 206 codes that were developed out of open coding and affinity clustering: (1) screening tools for identifying social determinants of health (SDOH) barriers after discharge, (2) active patient and caregiver engagement in the discharge process, (3) follow-up postdischarge phone calls, (4) patient comprehension of discharge education, (5) team-based readiness tools to assess patient readiness for safe discharge from the hospital, and (6) consistency across the care continuum.

On the basis of these 6 themes, we formalized the transition of care model—the socioeconomic screening, active patient engagement, follow-up, education, discharge readiness tool, and consistency (SAFEDC) model (I-MPACT transition of care model)—that future initiatives can adopt and use to improve patient experience in care transitions (see Multimedia Appendix 4).

### Theme 1: SDOH Screening—Screening Tools to Identify Specific Health or Socioeconomic Barriers After Discharge

One of the critical factors identified for transitions of care improvement is the need for tools to better identify SDOH factors that affect patients after discharge. The lack of such screening tools was identified as a barrier to optimizing tailored care for patients. For example, targeted interventions for patients with specific health conditions are difficult to conduct if some of these conditions are not appropriately identified until after hospital discharge. One such intervention developed by workshop participants involves a multilevel, team-based screening system that captures feedback from multiple clinicians at different points along the care continuum. The screening system would help identify patients with lower socioeconomic status and ensure that a patient has the means to obtain medications, adhere to their prescribed treatment plan, and make it to their follow-up appointments after discharge. Another intervention highlights the importance of screening all patients who transfer to a skilled nursing facility with a standardized SDOH screening tool before discharge. Once the standardized SDOH screening is completed, the hospital-based care coordination team can determine whether patients need further assistance. If a need for assistance is identified, care coordinators will communicate with each facility independently based on the patient’s needs so that they can offer aid with the necessary resources (eg, options for medications). Screened information and identified needs can be integrated into the electronic health record (eg, Epic) and transferred to respective care coordination programs or other hospitals. See Multimedia Appendix 5 for an example of the SDOH Assessment Screening Tool that was implemented by one of the hospitals participating in the workshop.

### Theme 2: Active Patient Engagement—Active Patient and Caregiver Engagement in the Discharge Process

Another barrier identified in care transitions is the lack of patient and caregiver involvement in the discharge process. Participants noted that the current discharge process often does not provide enough options for patients and their caregivers to communicate their specific health care needs with providers after hospital discharge. The lack of active patient and caregiver involvement negatively affects the patient’s care transition experience.

To effectively engage patients and caregivers across the care continuum, participants highlighted the necessity of creating explicit systems to better allow patients to engage in their care. The use of practical tools for providers to better understand and support patients’ specific health-related goals and motivations was theorized to lead to a more effective and patient-tailored care plan. For example, providers pointed out the need for more patient-friendly communication tools that can better empower patients to improve medication adherence when medication noncompliance has been identified. In addition, a patient-tailored tool could invite and empower patients to engage in advanced care planning conversations at various stages of their illness and better equip clinicians to deliver care personalized to meet individual patients’ needs. During the workshop, providers shared that when they perceive barriers to patient or caregiver engagement, the providers feel less equipped, are less motivated, and perceive that there is more bias when offering additional support to their patients at the time of discharge. A standard protocol for patient engagement may help reduce these barriers and better engage patients and caregivers in patient-centered care plans.

In addition, participants suggested technology-mediated interventions that could facilitate goal planning and tracking. One of the examples presented by the participants was to provide ways of generating personal goals and regularly document them in their heart failure symptom tracker (eg, Heart Smart Calendar and My Heart Failure Action Plan). See Multimedia Appendix 5 for an example of an intervention aimed at promoting active patient and caregiver engagement during the discharge process.

### Theme 3: Follow-up—Improving Postdischarge Follow-up

A prerequisite for participation in the I-MPACT workshops was a commitment to increase rates of the 7-day posthospital follow-up for patients. In addition to improving the postdischarge follow-up, participants also identified the need for more complete postdischarge follow-up protocols. Although many hospitals noted that they often call patients after discharge, it was determined that current phone calls are often unstructured and uncoordinated between the different organizations that provide posthospital care. The lack of an integrated call process between the hospital and POs results in fragmentation of care, creating difficulties in ensuring whether patients receive appropriate follow-up assistance, as well as important information (eg, follow-up clinic appointment schedule) from their care providers.

To alleviate this problem, our participants emphasized the need to have clear goals, protocols, and improved structures for follow-up phone calls. For example, the participants suggested that there would be value in having a standard approach to close follow-up phone calls with patients in both the immediate postdischarge period (eg, within 2 days of discharge) and the early postdischarge period (eg, within a week). See Multimedia Appendix 5 for an example of a structured postdischarge phone call, which was implemented by one of the hospitals that participated in the workshop.

### Theme 4: Education—Patient’s Comprehension of Discharge Education

We also identified that a patient’s comprehension of discharge education was a potential factor that affects the effectiveness and quality of care transition. Workshop participants raised concerns that patients often do not comprehend the educational materials and are given an extensive amount of information (eg, precautions, safety protocols, and medication instructions) in later phases of their hospital stay and at the time of discharge. Patients often receive lengthy handbooks and materials to review after they leave; however, the patients participating in the workshop stated that these materials are often unread. Participants noted that patient education materials could be ineffective as they are often generic and not tailored to each patient’s individual circumstances.

It was further noted that current discharge processes do not involve effective methods to ensure that patients comprehend the information that they are given. Workshop participants pointed out the need for effective strategies to deliver core discharge information, such as medication teaching, to patients and their caregivers earlier during hospitalization. Examples of recommended interventions included applying teach-back methods with tailored tools and simplified educational materials. Re-educating nursing staff about teach-back methods and clarifying caregivers’ roles and responsibilities for patient education were proposed as ways of improving the effectiveness and quality of discharge education. Multimedia Appendix 5 provides an example of an intervention to promote patient comprehension of discharge education—a simplified, patient-centered education that was implemented by one of the hospitals participating in the workshop.

### Theme 5: Discharge Readiness—Team-Based Tools That Assess Readiness for Safe Discharge

The findings highlight the importance of having a standardized, multidisciplinary discharge readiness assessment, in which all team members can provide input and receive feedback regarding the patient’s readiness for safe hospital discharge. The participants agreed that, currently, there is limited availability of such a tool; however, its creation and use would allow the multidisciplinary team to better understand and communicate discharge readiness.

Participants discussed the value of a team-based perspective to determine whether the patient is ready for discharge and a multidisciplinary approach for how to best minimize risk and improve safety for the patient. Participants hypothesized that using a team-based readiness tool would improve communication, optimize workflow, and allow an improved multidisciplinary approach to identifying potential barriers and the action plans needed to overcome them. By prioritizing and adjusting the workload for a multidisciplinary team, a team-based readiness assessment tool could assist in the evaluation of safe discharge. See Multimedia Appendix 5 for an example of implementing a team-based discharge readiness assessment tool that was implemented by one of the hospitals.

### Theme 6: Consistency—Consistent Transition of Care Processes Across the Care Continuum

One of the most frequently mentioned themes across the clusters was that patients and caregivers experience a lack of consistency as they move from one episode of care to the next. The workshop participants noted that inconsistency could significantly affect patients’ experiences. Participants shared how uncoordinated and inconsistent information received from different providers negatively affected a patient’s comprehension and interfered with their ability to actively engage and participate in their own care. Examples of inconsistent care included conflicting information from the provider (eg, discrepancies in discharge instructions), uncoordinated phone calls from multiple providers after discharge, incongruent follow-up appointments, and incomplete information or misinformation from different clinics (eg, incorrect physician names). In addition, the multitude of inaccessible electronic health records across the continuum of care prevents patients from accessing important records and impedes their awareness and comprehension. These findings challenged our participants to consider the use of a standard communication process between providers and patients and the implementation of active collaboration across multidisciplinary teams to proactively plan discharge.

The proposed intervention involved a hospital notifying the PO that their patient had been admitted to the hospital and the PO then providing a longitudinal care management program for the patient to follow for 90 days after discharge. Another intervention involved a care management program for all patients transferred from the hospital to a skilled nursing facility. See Multimedia Appendix 5 for an example of an intervention aimed at improving consistency across the care continuum.

### Intervention Sustainability Follow-up

In this section, we summarize the current status of intervention implementation in hospitals during the postworkshop phase. Initial interventions were conducted during or shortly after the workshop. Furthermore, each cluster updated the QI log biannually, recording how the intervention was adopted, modified, or maintained. Interventions could be adopted depending on the hospital’s resources, patient needs, and what could be sustainable and widely disseminated for that cluster’s target population. To receive points in the program, the cluster must meet a certain threshold for intervention dissemination. Each hospital generated the most feasible care transition intervention tailored to the needs of its health system during the postworkshop period based on the 6 themes. Examples of practical interventions included a 90-day PO care management enrollment program, advanced care planning, and follow-up phone calls after discharge (see Multimedia Appendix 4 for example interventions). Although our primary aim was to present the impact of the participatory design process on brainstorming interventions for care transition rather than longitudinally following up on the implementation process, we also captured postworkshop progress to assess the sustainability of these ideas. Each cluster updated its progress in a biannual progress report called the QI log. We used the August 2020 progress report to gauge the intervention’s sustainability.

Of the 24 hospitals that joined the I-MPACT and participated in the workshop, 5 (21%) were not included in the study as 3 (60%) hospitals did not conduct an observation to generate a patient journey map, and the other 2 (40%) hospitals opted out of the I-MPACT before August 2020. As a result, 79% (19/24) of hospitals remained in the project for follow-up and were analyzed in this study. Each of the 19 hospitals generated and implemented at least two feasible transition of care interventions, resulting in a total of 47 original interventions. From the August 2020 progress report, there were 47 total interventions, of which 24 (51%) were original interventions generated during or after the workshop, 10 (21%) interventions changed but were related to the originally proposed interventions, and 13 (28%) interventions were new interventions that hospitals generated by themselves during the postworkshop period. Of the 19 clusters that participated, 13 (68%) sustained at least one of their original interventions, 2 (11%) sustained all of their original interventions, and 4 (21%) did not sustain any of their original interventions. In summary, it was found that most clusters currently implemented the same interventions or those incorporated themes similar to the original interventions.

## Discussion

### Principal Findings

This study aimed to explore the effectiveness of the collaborative design approach in creating transition of care interventions that can potentially improve the hospital discharge experience for patients and reduce adverse outcomes, including readmissions. Through our qualitative analysis, six primary themes that facilitate patient care transitions emerged: (1) screening tools for identifying SDOH barriers after discharge, (2) active patient and caregiver engagement in the discharge process, (3) follow-up postdischarge phone calls, (4) patient comprehension of discharge education, (5) team-based readiness tools that can assess a patient’s readiness for safe hospital discharge, and (6) consistency across the care continuum. On the basis of these themes, each hospital developed tailored interventions to improve patients’ care transition experiences in their hospitals. During the follow-up period, all hospitals implemented at least one intervention originating from the initial workshop.

The SAFEDC model can be applied to improve patients’ experiences during care transitions. Our findings support the usefulness of gathering stakeholders from a local hospital and involving the patient perspective to help identify local gaps associated with transitions of care.

By presenting how collaborative efforts can be transformed into practical interventions, our study makes two main contributions: (1) generated insights and useful innovations for transition of care interventions by forming multidisciplinary teams involving patients, hospital systems, and outpatient systems across the care continuum and (2) established the usefulness of the participatory design approach in the context of health care quality improvement.

### Implications for Transitional Care Interventions

Given the number of health care interventions developed by workshop participants to improve care transitions, our study contributes to the current literature by highlighting the types of interventions that have been developed to improve care transitions and reduce hospital readmission rates. Previous studies in the context of care transition often involved 1 to 2 institutions as the target site [9,34,35]. However, in this study, close to 300 people from 19 health systems generated major concerns that should be considered when designing transition of care interventions. This study is an example of a large-scale project addressing care transitions using a participatory design approach. On the basis of workshop participants’ insights and interventions, we established 6 major themes that health systems could address when establishing transitional care interventions.

Patient-targeted approaches (eg, interventions directly involving the patient), provider-targeted approaches (eg, interventions aimed at better equipping the provider with information), and system-targeted approaches (eg, interventions aimed at improving care consistency) were generated. For example, 33% (2/6) of the important themes, including the team-based assessment of a patient’s readiness for safe discharge and screening to better identify vulnerable patients with particular conditions, particularly emphasized the provider role, which is historically less visible to patients. Alternatively, 3 themes were patient centered: active patient and caregiver engagement in the discharge process, postdischarge follow-up to ensure patients are on the right track, and patient comprehension of discharge education. The last theme highlights improving consistency across the care continuum, requires a more integrated view of the health system, and emphasizes the importance of cohesive work within hospitals and between health organizations. This study highlights the need for a multipronged approach to transition of care interventions.

These themes show that transitions of care should be regarded as an ongoing and continuous process rather than a series of intermittent events, which aligns with previous literature highlighting the importance of multidisciplinary, patient-centered approaches to care transitions that span the care continuum [36-39]. There is no one size fits all intervention that can facilitate care transitions for every individual or context. Depending on each hospital’s unique situation, including the available resources, targeted population, and prioritized problems, the specific details of an intervention should be carefully considered.

Using the SAFEDC model, future studies might propose simple collaborative activities or educational materials (eg, predesigned format to fill out and view multimedia content explaining the SAFEDC model) for health care institutions seeking to determine the six salient areas needing improvement within their specific context. For example, multiple stakeholders from health care institutions can apply the SAFEDC model to their situation and exchange perspectives to generate in-depth discussion.

### Sustainability of Participatory Design in the Health Care Environment

By highlighting the various roles of the stakeholders involved in transitions of care who developed sustainable interventions, this study shows the benefits of using a participatory design workshop in large-scale quality improvement efforts.

Implementing interventions within health care systems beyond the project timeline has been an important focus of recent studies on participatory design [40]. Although valuable insights and discussions were generated during the workshop, previous studies noted that many useful outcomes were minimally incorporated into the organization or community after the events because of the lack of follow-up and sustained methods of implementing outcomes [40,41]. Consequently, participatory design outcomes are often poorly diffused to the organization or community when a research project ends [40,41]. In complex social settings such as hospitals, limited use of available resources (eg, human resources and infrastructure) has become the main barrier that negatively affects the impact of participatory design recommendations. As we followed up with each hospital to understand their postworkshop practices, we shed light on the improved sustainability of participatory design outcomes.

We brought together various stakeholders of the health care environment, including patients and caregivers, and successfully generated transition of care interventions. We confirmed from our follow-up review that of 47 interventions implemented across 19 hospitals, 24 (51%) of the 47 interventions had remained the same over a period of approximately 3 years. As long-term outcomes (eg, 3 years) of participatory design efforts have been infrequently discussed in previous studies, our study demonstrates that participatory design as a sustained approach is capable of generating large-scale interventions that can be implemented in hospitals and health care systems.

### Limitations

Our study has some limitations. First, the lack of measurement tools to understand sustainability across hospitals was one of our limitations. Although we did our best to provide details of the intervention evaluation process across hospitals, this study does not offer an effective tool for the in-depth understanding of the long-term effectiveness of the intervention. Second, we did not collect sufficient information on why the clusters no longer used their interventions at certain points. Despite the specific number of clusters that continued with their original interventions, we have limited information on the specific factors that did not work in certain situations or contexts. Future studies should address these issues and generate contextual implications from long-term follow-ups using practical measurement tools.

### Conclusions

We conducted observations aimed at understanding patient discharge experiences and held a participatory design workshop to gather rich end user perspectives of stakeholders, including health care professionals and patients. Patient journey maps were used as useful triggers for conversations among various stakeholders during the workshop. On the basis of these findings, we proposed the use of a transition of care model, SAFEDC, in which future research and practices can be used to improve patient experiences in care transitions. As the study did not examine the direct opinions of workshop participants or intervention users, future studies may gain additional insights by following up with intervention user experiences, real use cases, and factors that may aid in understanding the facilitators and challenges for each implemented intervention. Given the increasing interest in quality improvement through patient-centered approaches to designing health care interventions, this study demonstrates ways of enhancing care transitions through user-centered interventions.

## Appendix

Multimedia Appendix 1 Patient journey map generated from the observation study.

Multimedia Appendix 2 Participatory design workshop held in February 2017.

Multimedia Appendix 3 Discharge timeline creation during the workshop.

Multimedia Appendix 4 Socioeconomic screening, active patient engagement, follow-up, education, discharge readiness tool, and consistency model (Integrated Michigan Patient-centered Alliance in Care Transitions transition of care model).

Multimedia Appendix 5 Example interventions.

## Abbreviations

CQI: Collaborative Quality Initiative
HRRP: Hospital Readmission Reduction Program
I-MPACT: Integrated Michigan Patient-Centered Alliance in Care Transitions
PO: physician organization
QI: quality initiative
SAFEDC: socioeconomic screening, active patient engagement, follow-up, education, discharge readiness tool, and consistency
SDOH: social determinants of health
