# Supplementary material for: Development of the Socioeconomic Screening, Active Engagement, Follow-up, Education, Discharge Readiness, and Consistency (SAFEDC) Model for Improving Transitions of Care: Participatory Design
Source: JMIR Form Res. 2022 Apr 12;6(4):e31277. doi: 10.2196/31277 (PMC9044161; doi:10.2196/31277)
Supplement: Multimedia Appendix 1 [file formative_v6i4e31277_app1.docx]

**Multimedia Appendix 1**

Patient journey map generated from the observation

[PDF, 311,173 bytes]


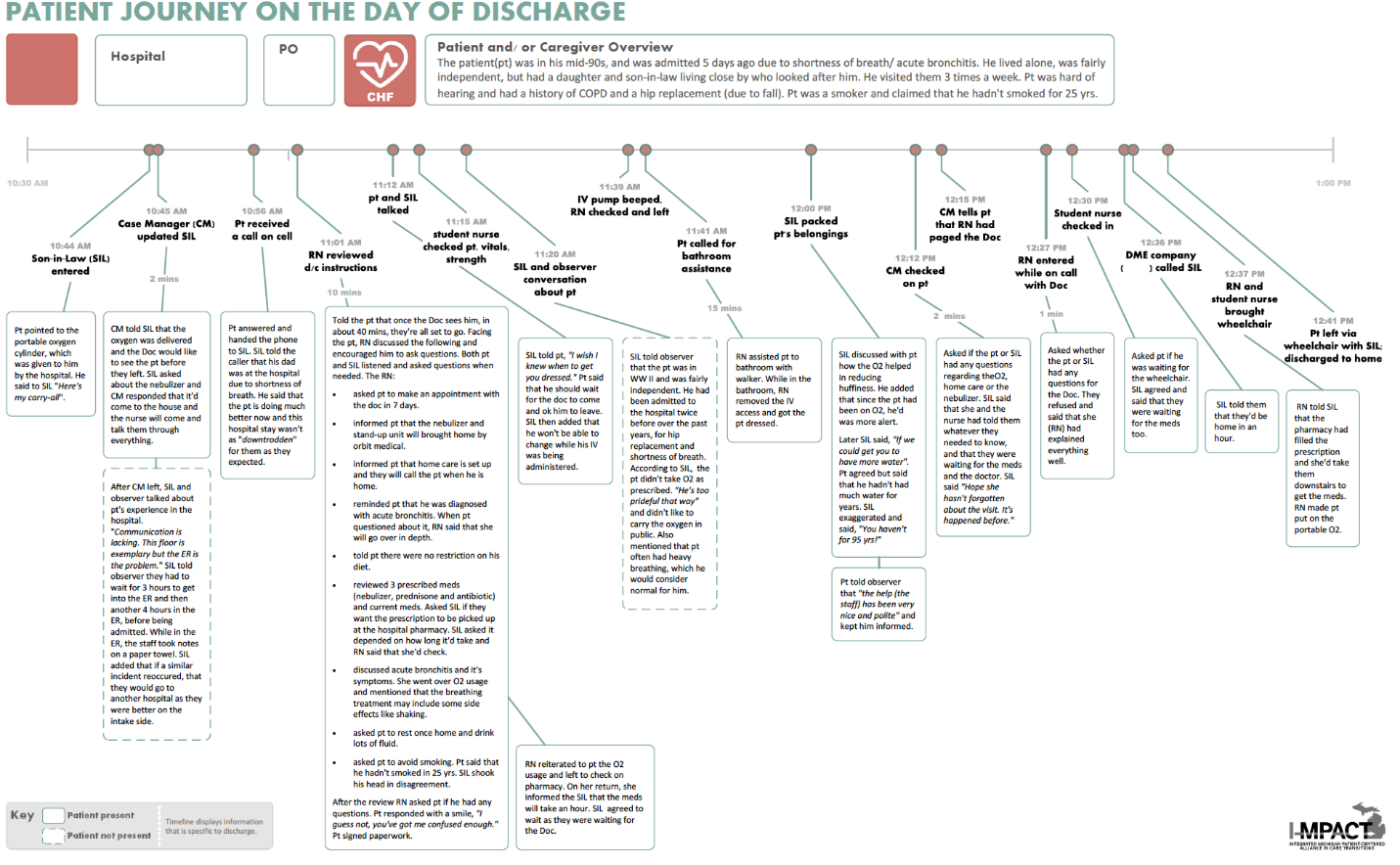


***Participatory design workshop***

When each cluster joined I-MPACT, they were required to attend a participatory design workshop. This all-day kick-off event was intended to bring a cluster together to discuss local transition of care data and share ideas and insights around the patient’s perspective from various points along the care continuum. Clusters were asked to use this workshop to generate ideas about what problems the local cluster would target during their participation in I-MPACT over the next several years to improve transitions of care and patient experiences. To have a more collaborative environment and draw rich insights from multiple stakeholders, each kick-off was organized to create a collaborative environment, draw rich insight from multiple stakeholders, and to highlight the patient perspective.

Five participatory design workshops were held between 2016 to 2018 (see Table 1). With each cluster attending this kick-off event, the aim was to foster discussion where participants could share multiple perspectives, capture the full scope of the care transition process, and generate practical design implications for improvement in care transitions. Each workshop had approximately 40 to 65 participants. During the full-day workshops, participants consisting of patients, caregivers, physicians, nurses, administrators, and designers worked in groups of seven to nine people within their cluster to generate problem statement(s) and begin initial discussions regarding interventions. Each cluster cohort participated in one workshop.

At each kickoff workshop, the participants were provided with patient journey maps created from the observation of a patient discharged from the hospital within their cluster (see Appendix 2). The details shared from each of the 26 observed discharges focused on the transition of care discussion from the patient’s perspective in an effort to enable participants to gain a better perspective on problems that may otherwise have gone unnoticed by others. Participants also shared their individual experiences regarding the care transition, which contributed to a shared understanding of the entire discharge process.

After reviewing the patient journey map and initial discussions, participants were asked to collaboratively generate major problem areas that they aimed to improve upon through transitions of care interventions. Inspired by the service blueprint mapping in service design,[32] we encouraged participants to create their own hospital’s discharge timeline that detailed core activities of a typical patient’s care transition. This process enabled the participants to create a broader representation of the discharge process and consider major barriers and details that impact a patient’s experience during the care transition (see Appendix 3). During this process, each group was able to identify barriers, draft problem statements, and generate potential design interventions aimed at improving patient experiences throughout the care transition. If brainstorming was not completed during the workshop, each hospital finalized their brainstormed ideas for transitional care interventions during the post-workshop phase. As a result, 47 original interventions were generated with the aim of facilitating and improving care transitions. Each cluster was required to update their progress in a biannual report called the Quality Initiative (QI) log, and the August 2020 report was used to assess the status of their interventions. In the Results section, we provide an overview of post-workshop progress to gauge the sustainability of these interventions generated.

**Table 1. Study Cohorts and Their Kickoff Dates**

| **COHORT** | **CLUSTERS INVOLVED IN THE COHORT** | **START DATE** |
| --- | --- | --- |
| Cohort 1 | 1 CHF and 1 SNF | Feb 2016 |
| Cohort 2 | 4 CHF | Sept 2016 |
| Cohort 3 | 1 SNF, 5 CHF | Feb 2017 |
| Cohort 4 | 2 COPD, 1 CHF | September 2017 |
| Cohort 5 | 1 CHF, 3 SNF | Sept 2018 |

COPD: Chronic obstructive pulmonary disease, CHF: Congestive heart failure, SNF: Skilled nursing facility
