# Supplementary figures and images for: Development of the Socioeconomic Screening, Active Engagement, Follow-up, Education, Discharge Readiness, and Consistency (SAFEDC) Model for Improving Transitions of Care: Participatory Design
Source: JMIR Form Res. 2022 Apr 12;6(4):e31277. doi: 10.2196/31277 (PMC9044161; doi:10.2196/31277)

**Multimedia Appendix 2**

Participatory design workshop held in February 2017

[JEPG, 36,670 bytes]


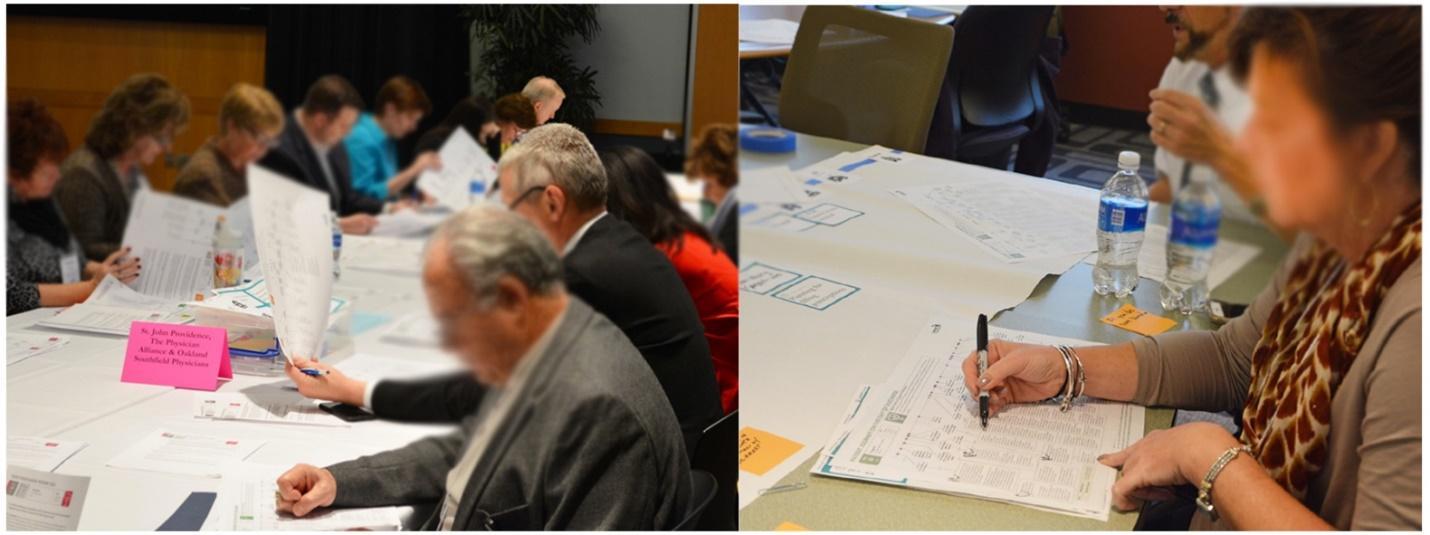

Supplement: Multimedia Appendix 2 [file formative_v6i4e31277_app2.docx]
