# Supplementary material for: Development of the Socioeconomic Screening, Active Engagement, Follow-up, Education, Discharge Readiness, and Consistency (SAFEDC) Model for Improving Transitions of Care: Participatory Design
Source: JMIR Form Res. 2022 Apr 12;6(4):e31277. doi: 10.2196/31277 (PMC9044161; doi:10.2196/31277)
Supplement: Multimedia Appendix 3 [file formative_v6i4e31277_app3.docx]

**Multimedia Appendix 3**

Discharge timeline creation during the workshop

[JEPG, 36,670 bytes ]


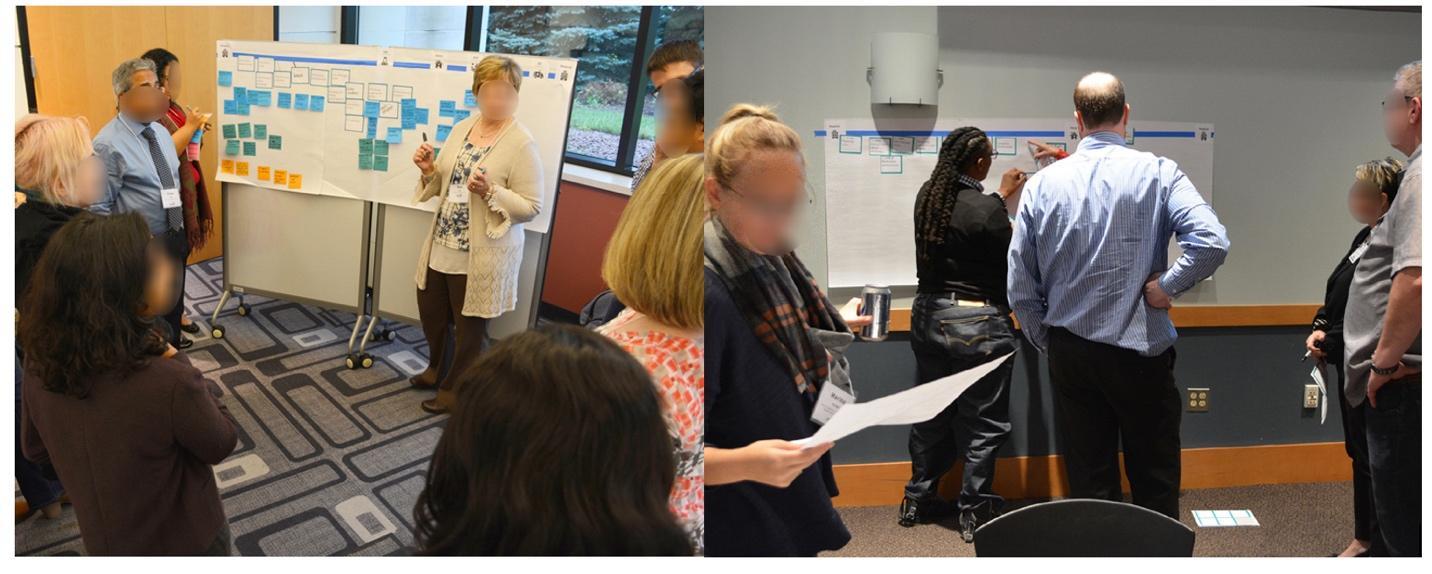


**Data Analysis**

We followed a constructivist grounded theory approach for data analysis [33]. Without a predetermined conceptual frame, we iteratively read the participants’ problem statements and identified commonly addressed themes in a collaborative matter. This methodology enabled the team to understand participants’ experiences and views on care transition rather than our views or preconceptions as healthcare researchers. To find the patterns of major discussion during the participatory design workshop, we analyzed problem statements and proposed interventions collected from 19 of the 24 hospitals after the workshop. Five hospitals were not included in the study because 3 hospitals did not conduct an observation to generate a patient journey map, and the other 2 hospitals opted out of I-MPACT before August 2020. The lead author performed open coding using NVivo Pro 12. While the data analysis was performed, the team discussed the general direction and primary focus to create a shared understanding. The team iteratively reviewed data to identify major themes for the open coding. This resulted in the following topic areas: (1) emerging obstacles during the discharge processes; (2) problem statements that represent the most significant and feasible areas for improving care transitions among identified issues; and (3) generated design opportunities that could be turned into practical interventions. With these themes, we aggregated problem statements until they did not overlap one another. This open coding yielded 206 codes, including 106 emerging problems of the care transition and 100 opportunities to contribute to interventions. Some examples of coded problems included "lack of patient's involvement in managing their health," and "lack of consistent scheduling of follow-up appointments." Opportunities for interventions included: "ensure that patient and family caregivers understand education" and "encourage patients to take control over their care." We used affinity clustering to identify the commonalities and hierarchy of the 206 codes. The lead author performed the affinity clustering based on the commonalities and relationships between the themes, identifying the most salient emerging themes. The results were presented to the rest of the team to resolve any lack of agreement on themes. The affinity diagramming resulted in themes with three different levels, which allowed the team to capture overarching themes that encompassed the lower-level themes. Themes at the third or lowest level include: "patients cannot afford prescriptions," "differences in the role of social workers and case managers at a hospital." Second-level themes included "lack of early communication among providers" and "documenting patients' goals." Top-level themes included "needs for screening tools" and "the importance of consistency." This iterative analysis allowed the team to identify themes that workshop participants deemed the most critical and recognized as areas needing significant transitions of care improvement.

## Results

**Overview**

Six major themes emerged from the 206 codes that were developed out of open coding and affinity clustering: (1) screening tools for identifying social determinants of health (SDOH) barriers after discharge; (2) active patient and caregiver engagement in the discharge process; (3) follow-up post-discharge phone calls; (4) patient comprehension of discharge education; (5) team-based readiness tools to assess patient readiness for safe discharge from hospital; and (6) consistency across the care continuum.

Based on these six themes, we formalized the transition of care model, SAFEDC (socioeconomic screening, active patient engagement, follow-up, education, discharge readiness tool, consistency) (I-MPACT transition of care model) that future initiatives can adopt and use to improve the patient’s experience in care transitions (see Appendix 4).
