# Supplementary material for: Development of the Socioeconomic Screening, Active Engagement, Follow-up, Education, Discharge Readiness, and Consistency (SAFEDC) Model for Improving Transitions of Care: Participatory Design
Source: JMIR Form Res. 2022 Apr 12;6(4):e31277. doi: 10.2196/31277 (PMC9044161; doi:10.2196/31277)
Supplement: Multimedia Appendix 4 [file formative_v6i4e31277_app4.docx]

**Multimedia Appendix 4**

SAFEDC model (I-MPACT transition of care model)

[PDF, 60,918 bytes]

**
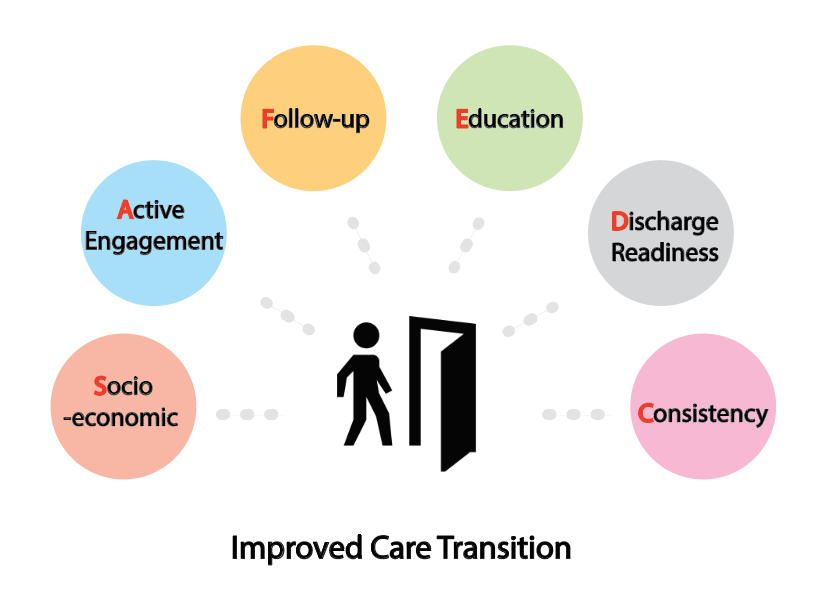
**

**Theme 1: Social Determinants of Health Screening**

***Screening tools to identify specific health or socioeconomic barriers after discharge***

One of the critical factors identified for transitions of care improvement is the need for tools to better identify SDOH factors that impact patients after discharge. The lack of such screening tools was identified as a barrier to optimizing tailored care for patients. For example, targeted interventions for patients with specific health conditions are difficult to carry out if some of these conditions are not appropriately identified until after hospital discharge. One such intervention developed by workshop participants involves a multi-level, team-based screening system that captures feedback from multiple clinicians at different points along the care continuum. The screening system would help identify patients with lower socioeconomic status and ensure a patient has the means to obtain medications, adhere to their prescribed treatment plan, and make it to follow-up appointment(s) after discharge. Another intervention highlights the importance of screening all patients who transfer to a skilled nursing facility (SNF) with a standardized SDOH screening tool prior to discharge. Once the standardized SDOH screen is completed, the hospital-based care coordination team can then determine whether patients need further assistance. If a need for assistance is identified, care coordinators will communicate with each facility independently based upon the patient's needs so that they can offer aid with necessary resources (eg, options for medications). Screened information and identified needs can be integrated into the electronic health record, eg, EPIC, and transferred to respective care coordination programs or other hospitals. See Appendix 5 for an example of the SDOH Assessment Screening Tool that was implemented by one of the hospitals participating in the workshop.

**Theme 2: Active Patient Engagement**

***Active patient and caregiver engagement in the discharge process***

Another barrier identified in care transitions is the lack of patient and caregiver involvement in the discharge process. Participants noted that the current discharge process often does not provide enough options for patients and their caregivers to communicate their specific healthcare needs with providers after hospital discharge. The lack of active patient and caregiver involvement negatively impacts the patient's care transition experience.

To engage patients and caregivers effectively across the care continuum, participants highlighted the necessity of creating explicit systems to better allow patients to engage in their care. The use of practical tools for providers to better understand and support the patient's specific health-related goals and motivations was theorized to lead to a more effective and patient-tailored care plan. For example, providers pointed out the need for more patient friendly communication tools that can better empower patients to improve medication adherence when medication noncompliance has been identified. Additionally, a patient tailored tool could invite and empower a patient to engage in advanced care planning (ACP) conversations at various stages of their illness and better equip clinicians to deliver care personalized to meet an individual patient’s needs. During the workshop, providers shared that when they perceive barriers to patient or caregiver engagement, the providers feel less equipped, are less motivated, and perceive there is more bias when offering additional support for their patients at the time of discharge. A standard protocol for patient engagement may help reduce these barriers and better engage patients and caregivers in a patient-centered care plan.

In addition, the participants suggested technology-mediated interventions that could facilitate goal planning and tracking. One of the examples presented by the participants was to provide ways to generate personal goals and regularly document them in their heart failure symptom tracker (eg, Heart Smart Calendar and My Heart Failure Action Plan). See Appendix 5 for an example of an intervention aiming to promote active patient and caregiver engagement in the discharge process.

**Theme 3: Follow-Up**

***Improving post-discharge follow-up***

A prerequisite for participation in the I-MPACT workshops was a commitment to increase rates of seven-day post hospital follow-up for patients. In addition to improving post-discharge follow-up, participants also identified the need for more complete post-discharge follow-up protocols. Although many hospitals noted they often call patients after discharge, it was determined that current phone calls are often unstructured and uncoordinated between the different organizations that provide post-hospital care. The lack of an integrated call process between the hospital and POs results in fragmentation of care, creating difficulties ensuring whether patients receive appropriate follow-up assistance, as well as important information (eg, follow-up clinic appointment schedule) from their care providers.

To alleviate this problem, our participants emphasized the need to have clear goals, protocols, and improved structure for follow-up phone calls. For example, the participants suggested there would be value in having a standard approach to close follow-up phone calls with patients in both the immediate post-discharge period (eg, within two days of discharge) and early post-discharge period (eg, within a week). See Appendix 5 for an example of a structured post-discharge phone call, which was implemented by one of the hospitals that participated in the workshop.

**Theme 4: Education**

***Patient's comprehension of discharge education***

We also identified that a patient's comprehension of discharge education was a potential factor that impacts the effectiveness and quality of the care transition. Workshop participants raised concerns that patients often do not comprehend the education materials and are often given an extensive amount of information (eg, precautions, safety protocols, medication instructions) in later phases of their hospital stay and often at the time of discharge. Patients often receive lengthy handbooks and materials to review after they leave, but the patients participating in the workshop stated these materials are often unread. Participants noted that patient education materials can be ineffective as they are often generic and are not tailored to each patient’s individual circumstances.

It was further noted that current discharge processes do not involve effective methods to ensure that patients comprehend information they are given. Workshop participants pointed out the need for effective strategies to deliver core discharge information, such as medication teaching, to patients and their caregivers earlier during hospitalization. Examples of recommended interventions included applying teach-back methods with tailored tools and simplified educational material. Re-educating nursing about teach-back methods and clarifying caregivers’ roles and responsibilities for patient education were proposed as ways to improve the effectiveness and quality of discharge education. Appendix 5 provides an example of an intervention to promote patient comprehension of discharge education: a simplified, patient-centered education that was implemented by one of the hospitals participating in the workshop.

**Theme 5: Discharge Readiness**

***Team-based tools that assess readiness for safe discharge***

Findings highlighted the importance of having a standardized, multidisciplinary discharge readiness assessment, in which all team members can provide input and receive feedback regarding the patient's readiness for safe hospital discharge. The participants agreed that, currently, there is limited availability of such a tool, but its creation and use would allow the multidisciplinary team to better understand and communicate discharge readiness.

Participants discussed the value of a team-based perspective to determine whether the patient is ready for discharge and a multidisciplinary approach for how to best minimize risk and improve safety for the patient. Participants hypothesized that using a team-based readiness tool would improve communication, optimize workflow, and allow an improved, multidisciplinary approach to identifying potential barriers and the action plans needed to overcome them. By prioritizing and adjusting the workload for a multidisciplinary team, a team-based readiness assessment tool could assist with the evaluation of safe discharge. See Appendix 5 for an example of implementing a team-based discharge readiness assessment tool that was implemented by one of the hospitals.

**Theme 6: Consistency**

***Consistent transition of care processes across the care continuum***

One of the most frequently mentioned themes across the clusters was that patients and caregivers experience a lack of consistency as they move from one episode of care to the next. The workshop participants noted that inconsistency can significantly affect patient experience. Participants shared how uncoordinated and inconsistent information received from different providers negatively impacted a patient's comprehension and interfered with a patient’s ability to actively engage and participate in their own care. Examples of inconsistent care include conflicting information from the provider (eg, discrepancies on discharge instructions), uncoordinated phone calls from multiple providers after discharge, incongruent follow-up appointments, and incomplete or misinformation from different clinics (eg, incorrect physician name). Also, the multitude of inaccessible electronic health records across the continuum of care prevent patients from accessing important records and impedes patient awareness and comprehension. These findings challenged our participants to consider the use of a standard communication process between providers and patients, and implementation of active collaboration across multidisciplinary teams to plan discharge proactively.

A proposed intervention involved a hospital notifying the PO that their patient has been admitted to the hospital and the PO then providing a longitudinal care management program for the patient to follow the patient for 90-days post-discharge. Another intervention involved a care management program for all patients transferred from the hospital to a skilled nursing facility. See Appendix 5 for an example of an intervention aimed at improving consistency across the care continuum.
