# Supplementary material for: Development of the Socioeconomic Screening, Active Engagement, Follow-up, Education, Discharge Readiness, and Consistency (SAFEDC) Model for Improving Transitions of Care: Participatory Design
Source: JMIR Form Res. 2022 Apr 12;6(4):e31277. doi: 10.2196/31277 (PMC9044161; doi:10.2196/31277)
Supplement: Multimedia Appendix 5 [file formative_v6i4e31277_app5.docx]

**Multimedia Appendix 5**

Example interventions

[docx, 20,227 bytes]

| **Theme** | **Example Interventions** |
| --- | --- |
| **Theme 1: Screening** | **SDOH Assessment.** Increase number of social determinants of health (SDOH) assessments completed on identified population. Patients receive care management across the continuum. Patients identified as high-risk through SDOH screening will have care management services tailored to those barriers identified. Those patients may require more interaction and more disciplines involved with their care to ensure appropriate interventions are completed. |
| **Theme 2: Active Engagement** | **Advanced Care Planning -** Patients have clear definitions of what their wishes are regarding end-of-life care, planning, and potential hospitalizations. A patient advocate/decision maker will be identified, and the patient/family/caregiver will understand their disease trajectory and the wishes of the patient if they are unable to speak for themselves. The conversation starts while the patient is in the hospital, and it can be completed with a signed document at the patient’s primary care office. |
| **Theme 3: Follow-Up** | **Bedside Visits and Phone Calls.** Complete bedside patient interviews, education, and follow-up calls with patients who are admitted with a chronic obstructive pulmonary disease (COPD) diagnosis. During the phone call, patients are asked to share their thoughts about why they needed to return to the emergency department (ED) and were admitted. They can ask about misunderstandings with medications, discharge instructions, appointments that were needed, and concerns that went unaddressed during their initial hospitalization. They can also talk about any new or worsening symptoms. They may receive advice on whether to go to the ED or see their primary care physician. |
| **Theme 4: Education** | **Teach-Back Materials.** Develop a task that reminds nurses to complete teach-back training on applicable nursing units. Re-educate nursing about congestive heart failure (CHF) teach-back techniques and implement an electronic tool that cues the nurse to complete education for each CHF patient. Patient and family caregivers confirm their understanding of education by utilizing teach-back techniques. |
| **Theme 5: Discharge Readiness** | **Discharge tool.** Develop a transition of care “discharge tool” map for discharge from hospital to a SNF for COPD patients from initial pilot site (Advantage Living). Transition of care “discharge tool” map from a hospital to a SNF. |
| **Theme 6: Consistency** | **Care Management.** All patients discharged from a hospital to a SNF will be enrolled into respective PO care management for follow-up. From a patient’s hospital discharge through their discharge from skilled care within a SNF, PO care management provides coordination of care with weekly monitoring and provides documentation to the primary care provider who follows the patient's discharge back into the community. Implementation of electronic and telephonic communication between inpatient care management and PO care management departments provides a daily disposition list of patients requiring care management in the SNF. |
